# Adrenomedullin blockade induces regression of tumor neovessels through interference with vascular endothelial-cadherin signalling

**DOI:** 10.18632/oncotarget.3167

**Published:** 2015-02-05

**Authors:** Ghizlane Khalfaoui-Bendriss, Nadège Dussault, Samantha Fernandez-Sauze, Caroline Berenguer-Daizé, Romain Sigaud, Christine Delfino, Mylène Cayol, Philippe Metellus, Olivier Chinot, Kamel Mabrouk, Pierre-Marie Martin, L'Houcine Ouafik

**Affiliations:** ^1^ Aix Marseille Université, CRO2, UMR_S 911, Faculté de Médecine, Marseille, France; ^2^ Inserm, U911-CRO2, Marseille, France; ^3^ Aix-Marseille Université, CNRS, UMR 7273, Institut de Chimie Radicalaire (ICR) Marseille, France; ^4^ AP-HM, CHU Nord, Service de Transfert d'Oncologie Biologique, Marseille, France

**Keywords:** adrenomedullin, tumor neovessels, angiogenesis, VE-cadherin, β-catenin

## Abstract

The cellular and molecular mechanisms by which adrenomedullin (AM) blockade suppresses tumor neovessels are not well defined. Herein, we show that AM blockade using anti-AM and anti-AM receptors antibodies targets vascular endothelial cells (ECs) and vascular smooth muscle cells (VSMCs), and induces regression of unstable nascent tumor neovessels. The underlying mechanism involved, and shown *in vitro* and *in vivo* in mice, is the disruption of the molecular engagement of the endothelial cell-specific junctional molecules vascular endothelial-cadherin (VE-cadherin)/β-catenin complex. AM blockade increases endothelial cell permeability by inhibiting cell-cell contacts predominantly through disruption of VE-cadherin/β-catenin/Akt signalling pathway, thereby leading to vascular collapse and regression of tumor neovessels. At a molecular level, we show that AM blockade induces tyrosine phosphorylation of VE-cadherin at a critical tyrosine, Tyr^731^, which is sufficient to prevent the binding of β-catenin to the cytoplasmic tail of VE-cadherin leading to the inhibition of cell barrier function. Furthermore, we demonstrate activation of Src kinase by phosphorylation on Tyr^416^, supporting a role of Src to phosphorylate Tyr^731^-VE-cadherin. In this model, Src inhibition impairs αAM and αAMR-induced Tyr^731^-VE-cadherin phosphorylation in a dose-dependent manner, indicating that Tyr^731^-VE-cadherin phosphorylation state is dependent on Src activation. We found that AM blockade induces β-catenin phosphorylation on Ser^33^/Ser^37^/Thr^41^ sites in both ECs and VSMCs both *in vitro* and *in vivo* in mice. These data suggest that AM blockade selectively induces regression of unstable tumor neovessels, through disruption of VE-cadherin signalling. Targeting AM system may present a novel therapeutic target to selectively disrupt assembly and induce regression of nascent tumor neovessels, without affecting normal stabilized vasculature.

## INTRODUCTION

Stability and functional assembly of tumor neovessels are governed by collaboration of multiple organ-specific cellular and angiogenic factors. Targeted genetic manipulation and antibody-mediated inhibition of angiogenic growth factors and their receptors in murine tumor models have resulted in identification of key angiogenic modulators that support tumor neoangiogenesis. Adrenomedullin (AM) is a widely distributed multifunctional peptide with properties ranging from inducing vasorelaxation to acting as a regulator of cellular growth [1]. AM binds to and mediates its activity through the G protein-coupled receptor calcitonin receptor-like receptor (CLR), with specificity for AM being conferred by the receptor activity modifying protein -2 (RAMP2) and -3 (RAMP3) [2]. The ability of CLR/RAMP2 and CLR/RAMP3 to respond with high affinity to AM implies the existence of two molecularly distinct AM receptors respectively referred to as AM_1_ and AM_2_ receptors [3]. The use of targeted mouse models clearly indicates that functional AM signalling is essential for embryonic survival. The genetic ablation of *Adm* [4, 5], *calcrl* [6], *Ramp2* [7–9] or the enzyme responsible for functional AM amidation, *peptidylglycine alpha-amidating monooxygenase* (*PAM*) [10] all result in midgestational lethality associated with severe interstitial edema and cardiovascular defects.

AM is widely expressed in a variety of tumor types [11] and was shown to be mitogenic for many human cancer cell lines *in vitro* [12]. Several *in vivo* studies have shown a regression of tumor neovessels and growth upon the treatment with neutralizing AM antibodies [13–15], AM receptor antagonist [16, 17], or AM receptor interference [18]. Therefore, understanding the mechanisms by which anti-AM antibody (αAM) and anti-AM receptors antibodies (αAMR) disrupt the integrity of tumor neovessels will identify the underlying biological mechanisms by which inhibitors of the AM/AMR disrupt integrated tumor vasculature.

An essential mediator factor that collaborates with many receptors to support the assembly of tumor neovessels is vascular endothelial cadherin (VE-cadherin), which is localized exclusively at specialized intercellular contact points of endothelium [19]. Cell-cell adhesion involves a variety of molecules, including the cadherin-catenin complex and the immunoglobulin superfamily member platelet endothelial cell adhesion molecule-1 (PECAM/CD31). The cadherins are single chain transmembrane polypeptides, which mediate homophilic, calcium-dependent adhesion and are specifically associated with the adherens junction region. VE-cadherin is involved in various aspects of vascular biology related to angiogenesis, most notably, endothelial cell assembly into tubular structures [20–22]. VE-cadherin null mouse embryos exhibit severely impaired assembly of vascular structures, leading to embryonic lethality at day E9.5, involving VE-cadherin as an important mediator in developmental angiogenesis [21]. Previous studies have shown that Src kinases play a general role in regulating cadherin function on a wide variety of cell types [23, 24]. β-catenin is a critical component of the cell-cell junction as it interacts with VE-cadherin to allow its attachment to actin microfilaments of cytoskeleton [25]. Therefore, β-catenin stabilizes the weak extracellular association between cadherin molecules [26].

The mechanism(s) by which αAM and αAMR selectively target and destabilize tumor neovessels is unknown. Here, we demonstrate that AM system blockade with αAM or αAMR disrupts endothelial cell junctions through rapid disengagement and inhibition of the VE-cadherin/β-catenin/Akt signalling pathway, leading to regression of tumor neovessels.

## RESULTS

We and others have shown that animals bearing xenografts tumors treated with αAM, αAMR, or AM antagonist AM_22–52_ reduced tumor growth with a clear tumor vascular disruption, suggesting that AM system might be crucial to stabilize neovessels during tumor growth as previously described [13–17]. At the molecular level, the mechanisms whereby inhibition of AM selectively target tumor neovessels are not known. To gain more insight into the mechanism(s) causing neovessels destabilization subsequently to αAM, αAMR, and AM_22–52_ treatment, we hypothesized that AM blockade might interfere through the endothelial cell junctions somehow to destabilize the tumor neovessels.

### αAM or αAMR induce endothelial cell death of tumor nascent vessels *in vivo*

**Figure 1:**
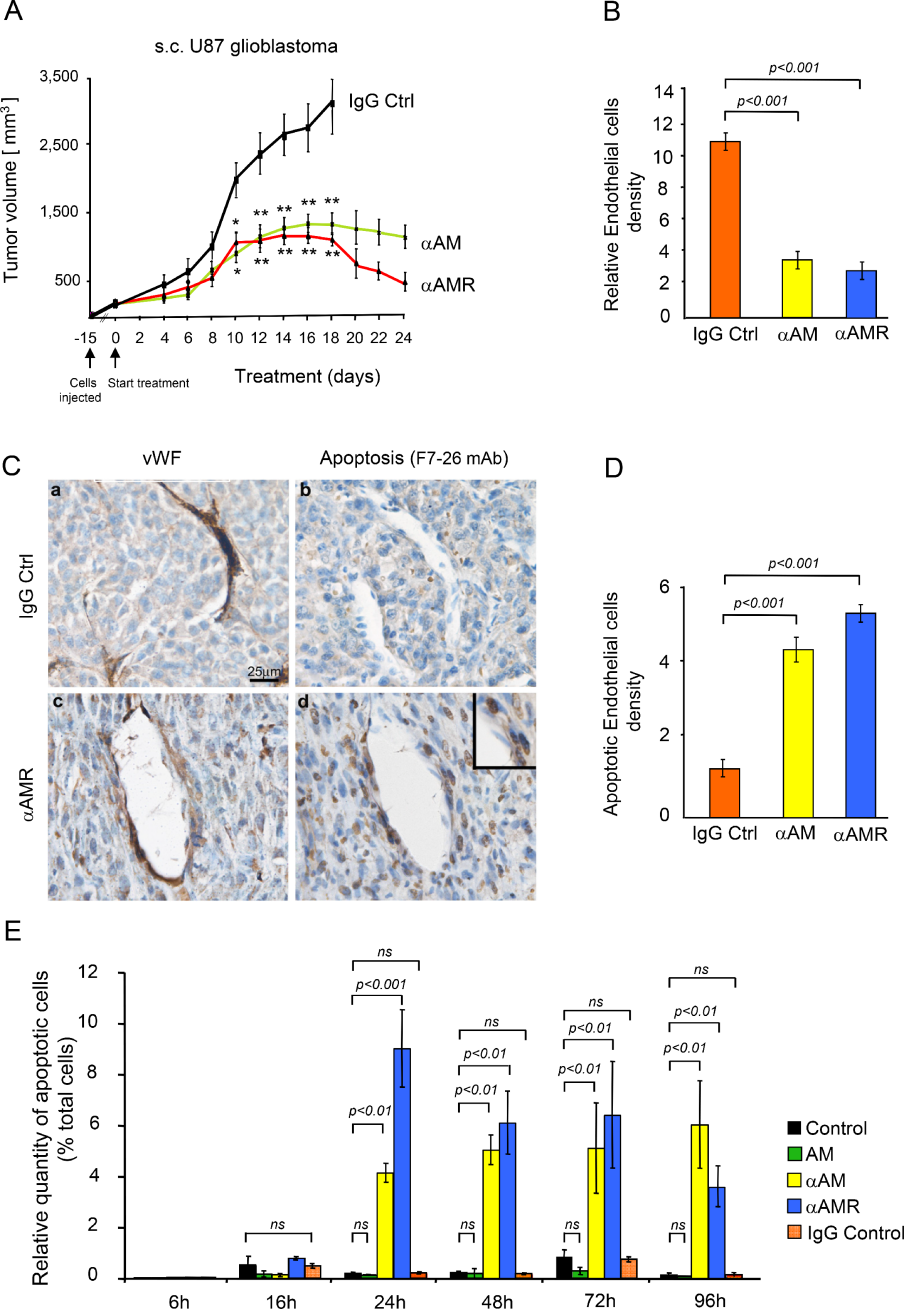
*In vivo* tumor analysis **(A)** measurements of tumor volume showed differences in growth of αAM (*n* = 10), αAMR (*n* = 10), and IgG-control (*n* = 6) treated-tumors during a 24 days time course. **(B)** quantitative assessment of cell density of cells that stained positive for CD31 was conducted through a microscope. MBF_Image J 1.43 U Software was used for analysis. Values are means ± SEM (*n* = 8). **(C)** AM blockade induces endothelial cell apoptosis. Control and αAM treated-tumors for 11 days were harvested and sections were immunostained for vWF and ssDNA. **(D)** quantitative assessment of endothelial cells undergoing apoptosis was determined by staining for ssDNA with F7–26 mAb. Values are means ± SEM (*n* = 6). **(E)** αAM and αAMR induce HUVECs death *in vitro*. HUVECs in presence of 2% FBS were incubated with IgG control (70 μg/ml), αAM (70 μg/ml), αAMR (70 μg/ml), or AM (10^−7^ M) for the indicated times, fixed and stained simultaneously with F7–26 mAb to detect ssDNA. The immunohistochemically positive cells that showed apoptotic bodies were quantified. Each point and bar represents mean ± SEM (*n* = 6) of three independent experiments. Where indicated, statistical analysis was performed with 1-way ANOVA followed by PLSD test, and the level of significance was set at *P* < 0.05.

Treatment with αAM and αAMR significantly inhibited the growth of s.c. U87 tumors compared with IgG control group (Figure 1A). After 18 days of treatment, a group of animals was sacrificed, and tumor size and vascularity were assessed. The immunohistochemical analysis of αAM and αAMR-treated tumors showed a clear decrease in microvessel density with 80% reduction of endothelial cells (Figure 1B). Both types of antibodies, αAM and αAMR, showed the same efficiency to induce a decrease of endothelial number (Figure 1B). These data support a role of AM in endothelial cells survival and/or recruitment to maintain a stabilized and functional endothelium during tumor growth as previously described [13–17]. The reduction of endothelial number might be due to apoptosis, impaired recruitment and/or inhibition of proliferation *in situ*. To gain insight into the mechanism(s) that destabilize neovessels and decrease endothelial number, we investigated the effect of αAM and αAMR on vascularization of U87 xenografts treated for 2, 6, 11, 16 and 25 days, to examine the integrity of vascular wall. The nuclear immunostaining with mAb F7–26 to stain ssDNA demonstrates apoptosis among U87 tumor cells as well as in cells located within the vascular lining (Figure 1C, d; *inset*). Staining with an antibody for vWF identified them as endothelial cells (Figure 1C, c). No apoptosis could be detected either among tumor cells or vascular endothelial cells in IgG control tumors (Figure 1C, a & b). These results indicate that endothelial cell death might be one of the causes responsible for the reduction of endothelial cells in αAM and αAMR-treated tumors. Incubation of HUVECs with αAM or αAMR induces endothelial cell death *in vitro* that is sustained up to 96 h (Figure 1E). On the contrary, treatment with AM protects HUVECs from death (Figure 1E), as previously reported [27, 28]. Taken together, these data support strongly that αAM and αAMR induce endothelial apoptosis *in vitro* as well as *in vivo* indicating AM as a potent survival factor for endothelial cells.

### The anti-vascular effects of αAM and αAMR are mediated through inhibition and disengagement of VE-cadherin/β-catenin function

**Figure 2:**
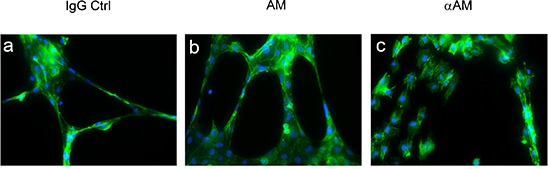
Morphogenetic activity of AM and αAM HUVECs (4 × 10^4^ cells/well) were seeded into Matrigel-precoated wells and cultured in low-serum conditions (0.5% FCS) in presence of IgG control (70 μg/ml) **(a)**, AM (10^−8^ M) **(b)**, or αAM (70 μg/ml) **(c)**. Photographs were taken 18 h later. Original magnification, × 20.

To explore the capacity of αAM and αAMR to inhibit angiogenesis, we measured their ability to disrupt the vascular structures formed by endothelial cells in an *in vitro* Matrigel assay. To this end, HUVECs that actively expressed AM and AM receptors and for which migration and invasion in Boyden assays *in vitro* were inhibited upon treatment with αAM or αAMR [29], were used as model to decipher the molecular mechanisms engaged by AM. Addition of AM (10^−7^ M) resulted in the formation of strong visible rings and cords of cells on growth factor-depleted Matrigel in the presence of 0.5% FCS (Figure 2b) when compared to non treated cells (Figure 2a). The incubation of HUVECs with αAM blocked tube formation and induced a clear cell-cell separation (Figure 2c) suggesting that AM promotes endothelial cell-cell interactions probably through modulation of junction proteins. Therefore, we tested the hypothesis that αAM and αAMR might interfere with VE-cadherin homophilic interactions.

**Figure 3:**
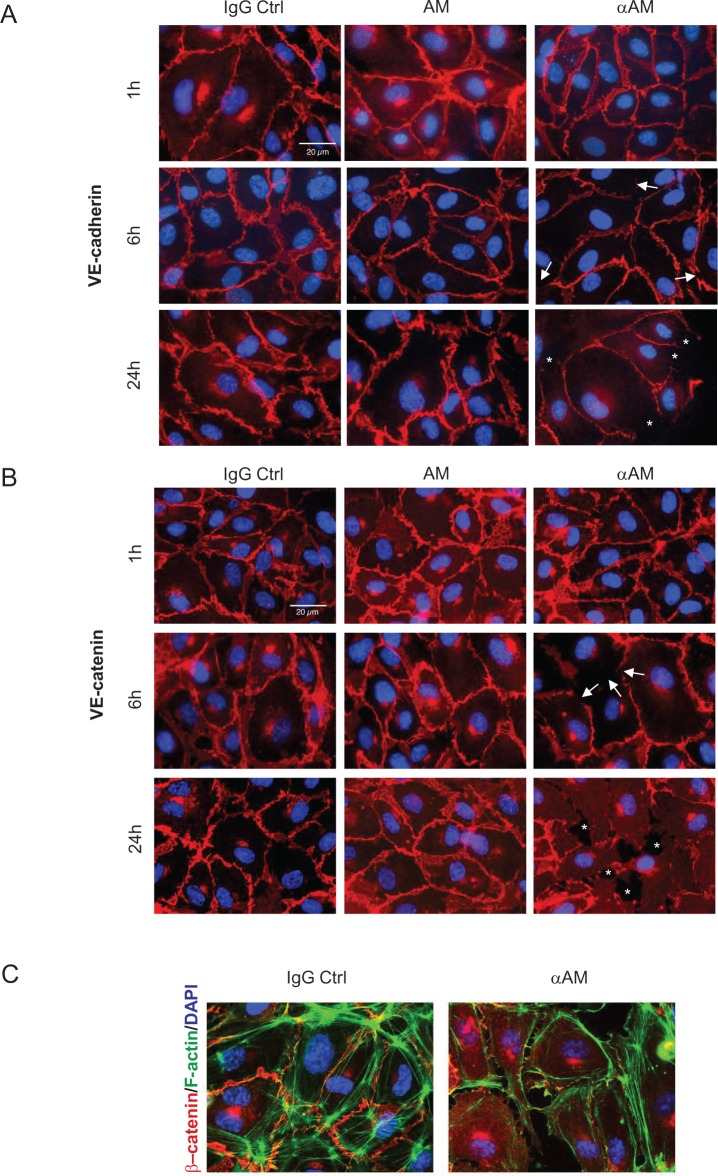
αAM and αAMR disengage VE-cadherin and disrupt β-catenin distribution in HUVECs *in vitro* **(A)** adherens junctions were assessed by immunofluorescence staining of clustered VE-cadherin molecules. The localization of VE-cadherin in confluent HUVECs monolayers was monitored during 24 h by microscopy. While VE-cadherin molecules are clustered in adherens junction of control and AM (10^−7^ M)-treated cells, addition of αAM (70 μg/ml) leads to redistribution of VE-cadherin disrupting cell-cell contacts that induce the apparition of holes between the cells (indicated by asterisks). DAPI-stained nuclei are in blue. **(B)** αAM disrupts the β-catenin distribution. The experiments and analyses for β-catenin were processed as described for VE-cadherin in A. DAPI-stained nuclei are in blue. **(C)** αAM treatment for 10 h caused a disengagement between cells, a reorganisation of actin fibers stained with phalloidin (green) that become localized around the cell body and β-catenin staining observed in the cytoplasm when compared to IgG-control treated cells. DAPI-stained nuclei are in blue.

Accordingly, HUVECs were incubated with AM (10^−7^ M), αAM (70 μg/ml), or IgG control (70 μg/ml). During the first hour of treatment, VE-cadherin was focally localized to the interjunctional region of endothelial cells (Figure 3A). After 6 h of treatment with αAM, the VE-cadherin staining at cell-cell contacts became very thin, and redistributed into a disorganized pattern (Figure 3A, arrow), which has been previously shown to be the hallmark of VE-cadherin disengagement [30]. However, at 24 h treatment, the redistributed VE-cadherin at the cell-cell contacts appeared to be associated with the partitioned and separated gaps between the endothelial cells suggesting a loss of intercellular contacts (Figure 3A, asterisks). The rapid initial VE-cadherin redistribution that occurred before cell retraction was concomitant with the redistribution of β-catenin, a signalling partner of VE-cadherin. In fact, staining for β-catenin showed a similar pattern at the same time points leading to the formation of separated gaps (Figure 3B, asterisks). Treatment with αAM induces a clear separation between endothelial cells accompanied with redistribution of actin fibres on the periphery and β-catenin staining localized at the cytoplasm (Figure 3C). The treatment with αAMR and AM_22–52_ demonstrated the same patterns as αAM (not shown). Incubation of human microvascular endothelial cells (HMEC) with αAM and αAMR *in vitro* induced cell-cell disruption between endothelial cells as demonstrated for HUVECs (Supplementary Figure S1). Therefore, we focused our attention on the mechanisms by which αAM and αAMR may exert their potent anti-vascular effects through destabilization of the VE-cadherin/β-catenin complex.

**Figure 4:**
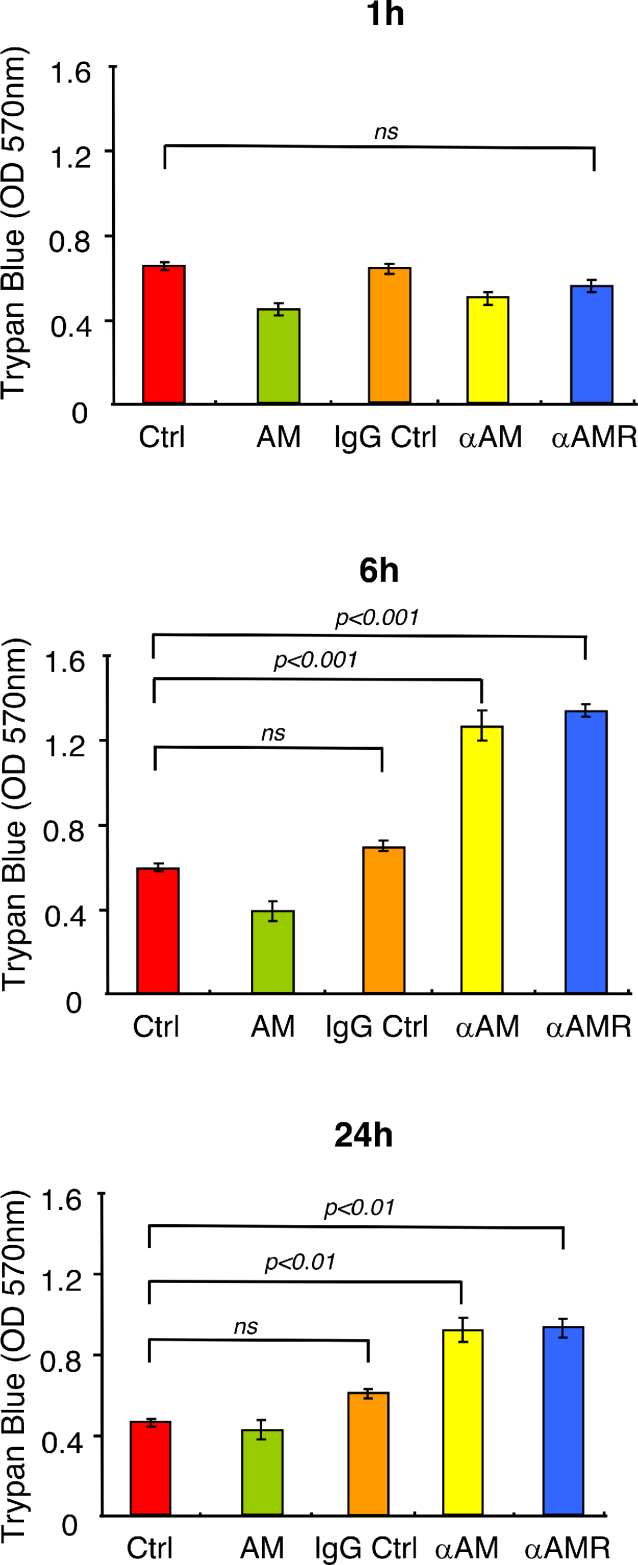
αAM and αAMR increase endothelial cell permeability *in vitro* Modification of endothelial cell permeability of αAM and αAMR-treated HUVEC monolayers was assessed as described in Materials and Methods at the indicated time points. Results of three independent experiments in triplicate are presented. Values are means ± SEM. Where indicated, statistical analysis was performed with 1-way ANOVA followed by PLSD test, and the level of significance was set at *P* < 0.05.

To determine whether αAM and αAMR induced loss of cell-cell interaction and disruption of VE-cadherin/β-catenin complex that would result in reduced monolayer integrity, we assessed the effect of αAM and αAMR on the integrity of intercellular junctions by measuring the permeability of a confluent HUVEC monolayer. Confluent monolayers of human endothelial cells cultivated on transwell filter inserts were treated with AM (10^−7^ M), αAM (70 μg/ml), αAMR (70 μg/ml), and IgG control (70 μg/ml). The permeability of the monolayer for Trypan Blue-BSA was determined at several time points by measuring the absorbance intensity of the medium in the lower compartment (Figure 4). In the absence of addition of AM (Ctrl), or presence of AM or IgG control, the level of Trypan Blue-BSA in the lower compartment was low and did not change during the time of experiments 1, 6 and 24 h (Figure 4). When the cells were treated one time with αAM or αAMR, we observed a significant increase in Trypan Blue-BSA permeability compared to control cells, which was highly significant after a delay of about 6 h and started to decrease by 24 h to reach the control values by 30 h of incubation (Figure 4). These results demonstrate that αAM and αAMR impair the endothelial cell barrier function through dysregulation of VE-cadherin homotypic interaction, leading to increased endothelial cell permeability.

**Figure 5:**
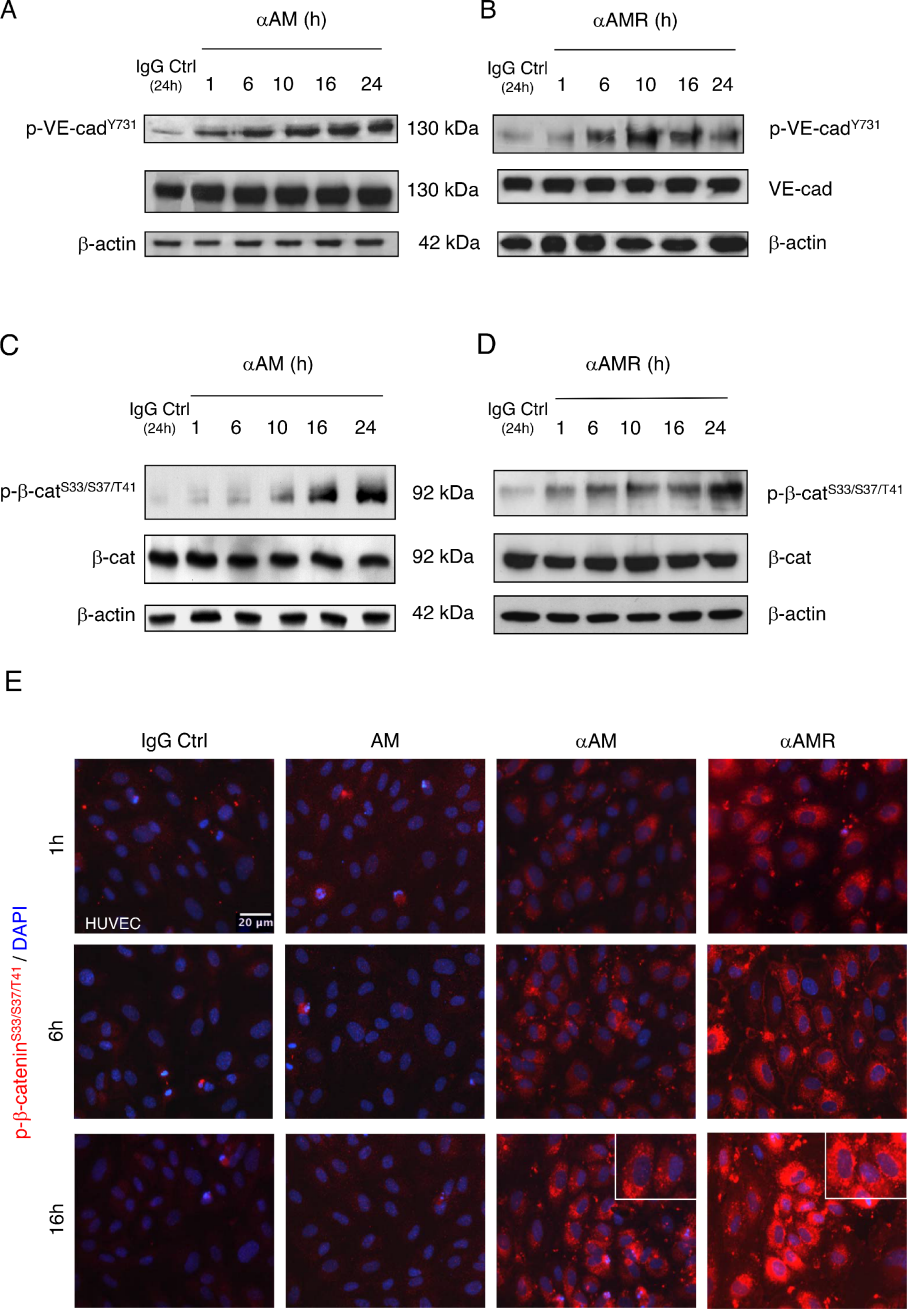
αAM and αAMR induce phosphorylation of Tyr^731^VE-cadherin and Ser^33^/Ser^37^/Thr^41^β-catenin in HUVECs *in vitro* **A** & **B**, αAM and αAMR induce Tyr^731^VE-cadherin phosphorylation in HUVECs in a time-dependent manner. **C** & **D**, αAM and αAMR induce Ser^33^/Ser^37^/Thr^41^ β-catenin phosphorylation in HUVECs in a time-dependent manner. No changes were observed in the total amount of the VE-cadherin (A, B) and β-catenin (C, D) suggesting that the increase in the phosphorylation patterns is independent of the changes in the overall protein expression. β-actin was used as a loading control. **(E)** Phospho-β-catenin is localized in the cytoplasm. The phospho-β-catenin Ser^33^/Ser^37^/Thr^41^ in confluent HUVECs monolayers was determined by fluorescent microscopy at different time points upon treatment with IgG-control (70 μg/ml), AM (10^−7^ M), αAM (70 μg/ml), and αAMR (70 μg/ml). DAPI-stained nuclei are in blue.

Previous studies have demonstrated that disruption of cell-cell junctions depends on intracellular kinases and/or phosphatases that regulate the phosphorylation state of cadherins and the cytosolic binding partners [31–34]. Accordingly, we sought to determine whether αAM or αAMR could interfere with VE-cadherin function, possibly through modulating VE-cadherin/β-catenin phosphorylation status. The mechanism by which αAM and αAMR modulates VE-cadherin/β-catenin function was further dissected in the presence or absence of 70 μg/ml of αAM or αAMR. The endothelial cell lysates were examined using anti-phosphoTyr^731^-VE-cadherin antibody, which recognizes VE-cadherin when it is phosphorylated on Tyr^731^, and anti-phospho-β-catenin Ser^33^/Ser^37^/Thr^41^ antibody, which recognizes β-catenin when it is phosphorylated at 1 or more of 3 specific sites, namely Ser^33^, Ser^37^ or Thr^41^. αAM and αAMR induced the phosphorylation of VE-cadherin on Tyr^731^ (Figures 5A, 5B) and β-catenin on Ser^33^, Ser^37^ or Thr^41^ (Figures 5C, 5D). No changes in the total amount of the VE-cadherin and β-catenin could be observed, suggesting that the increase of phosphorylation patterns are independent of the changes in the overall protein expression (Figures 5A–5D). The increase in phospho-β-catenin Ser^33^/Ser^37^/Thr^41^ was confirmed by immunostaining of αAM-treated endothelial cells and starts as early as 1 h (Figure 5E) and 16 h post-treatment demonstrates that the phospho-β-catenin reside specifically in the cytoplasm and is not translocated into the nucleus (Figure 5E, *inset*). These findings suggest that αAM and αAMR may interfere with β-catenin function, since phosphorylation of serine/threonine residues in the N-terminal region of β-catenin targets the protein for ubiquitination and subsequent proteasomal degradation [35, 36]. These data indicate that αAM or αAMR destabilizes the endothelial cell-cell junctions by promoting the phosphorylation of VE-cadherin and β-catenin which results in the loss of anchorage of endothelial cells and subsequently the disruption with its near cell partners.

### Src inhibitor impaired αAM and αAMR-induced VE-cadherin phosphorylation

**Figure 6:**
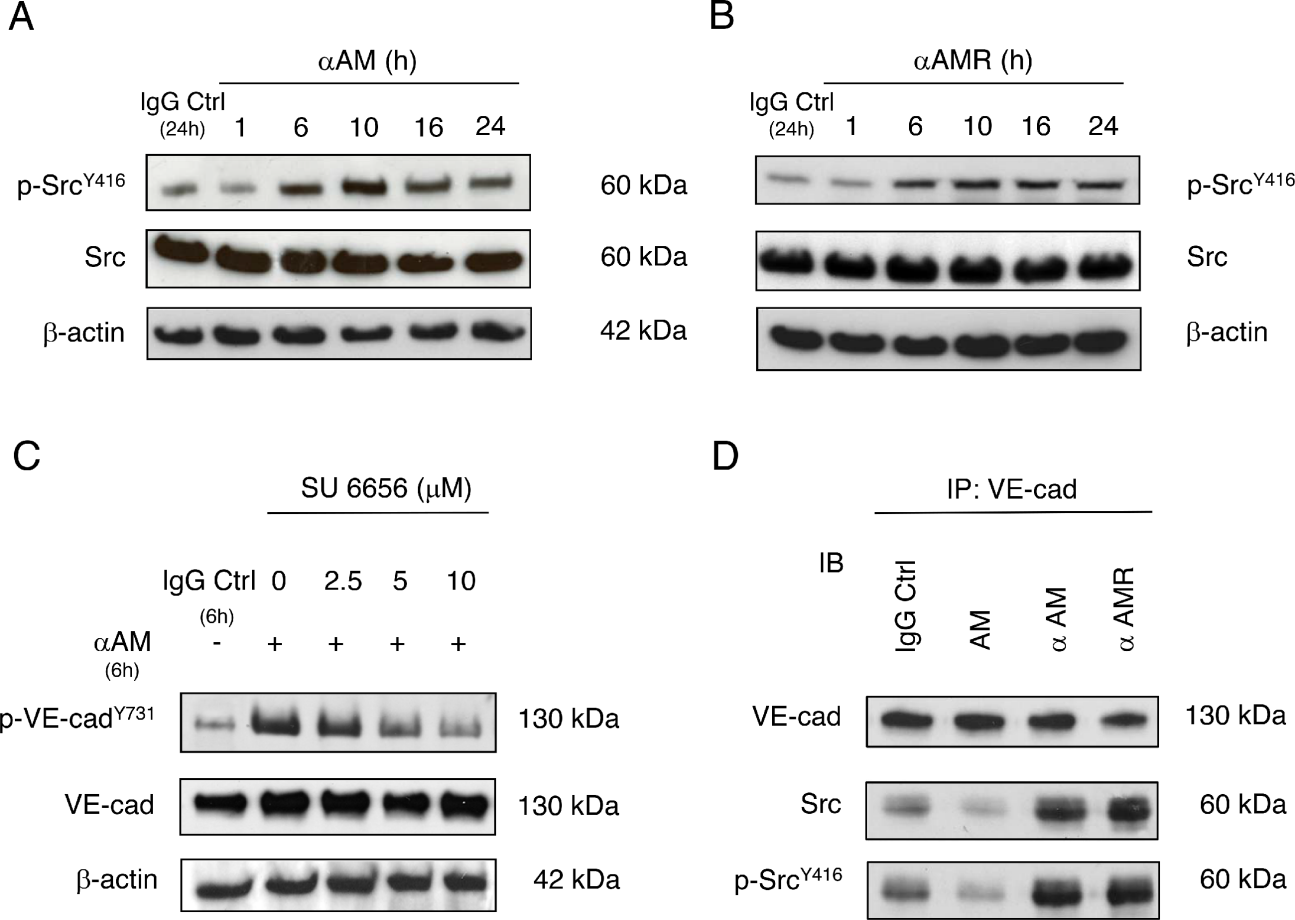
αAM and αAMR induce phosphorylation of Tyr^416^Src *in vitro* **(A & B)** Src becomes phosphorylated on Tyr^416^ in response to αAM and αAMR treatment. αAM (70 μg/ml) **(A)** and αAMR (70 μg/ml) **(B)** increased Tyr^416^ Src phosphorylation in HUVECs in a time-dependent manner. β-actin was used as a loading control. **(C)** Src inhibitor impaired αAM-induced VE-cadherin phosphorylation. HUVECs were preincubated with increasing concentrations of SU6656 as indicated and then treated with αAM (70 μg/ml) for 6 h. VE-cadherin and pY^731^-VE-cadherin were detected by Western blotting. β-actin was used as a loading control. **(D)** αAM and αAMR promote VE-cadherin/Src association. VE-cadherin was immunoprecipitated from HUVECs treated with IgG-control (70 μg/ml), AM (10^−7^ M), αAMR (70 μg/ml), and αAM (70 μg/ml). Immunoblotting was used to reveal VE-cadherin, Src and pY^416^-Src.

Previous studies suggest that Src kinases play a significant role in cadherin regulation [20, 37]. To determine whether phosphorylation of the VE-cadherin Tyr^731^ can be mediated by Src, we assessed the effects of αAM and αAMR on the phosphorylation of Src. The data demonstrate that Src was phosphorylated on Tyr^416^ in response to αAM and αAMR treatment revealing the strong control of AM on Src kinases (Figures 6A, 6B). No increase in total Src protein was observed, suggesting that the phosphorylation patterns are independent of the changes in the overall protein expression (Figures 6A, 6B). When HUVECs were pre-treated with Src inhibitor, SU6656 as indicated, αAM-induced VE-cadherin phosphorylation after 6 h was inhibited in a dose-dependent manner (Figure 6C). The same data were obtained with αAMR (not shown). We conclude that Src is required for αAM and αAMR-induced VE-cadherin phosphorylation on Tyr^731^. Furthermore, we demonstrate that αAM and αAMR promote Src-VE-cadherin association, meanwhile AM decreases the Src-VE-cadherin association (Figure 6D). VE-cadherin immunoprecipitates from AM, αAM, αAMR, and IgG-control-treated HUVECs were immunoblotted with anti-Src antibody. The presence of Src was detected in each immunoprecipitate and demonstrates a clear increase of Src association to VE-cadherin in αAM and αAMR-treated HUVECs when compared to IgG-control (Figure 6D). In contrast, treatment with AM reduces VE-cadherin/Src association (Figure 6D). These findings reveal that AM blockade with αAM or αAMR activates Src kinase and promotes VE-cadherin/Src association leading to the pTyr^731^VE-cadherin and thus may account for the well known role that Src kinases play in VE-cadherin-mediated cell-cell junctional activity [23].

### AM blockade decreases the phosphorylation of Akt Ser^473^

**Figure 7:**
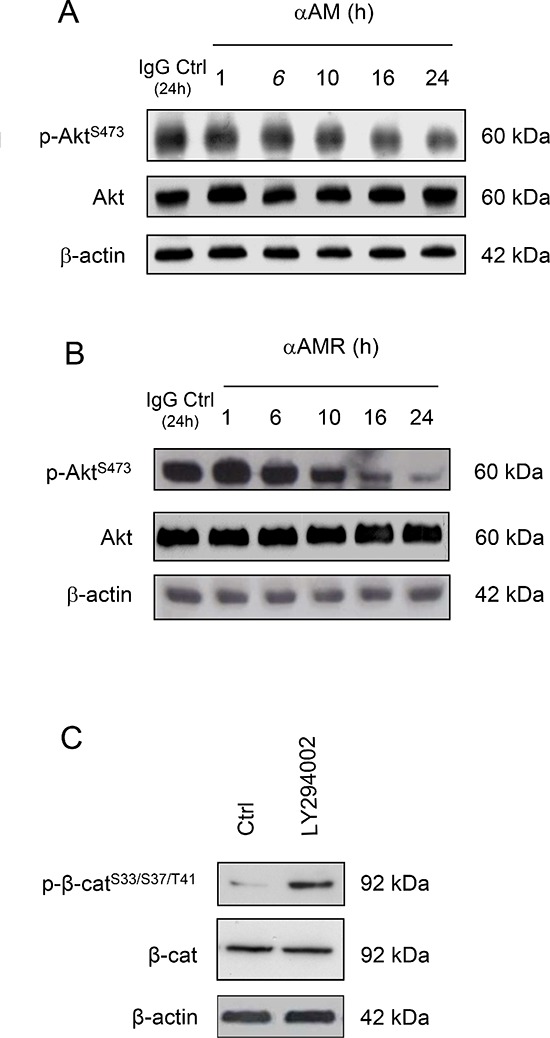
αAM and αAMR decrease phosphorylation of Ser^473^Akt *in vitro* **(A & B)** αAM (70 μg/ml) and αAMR (70 μg/ml) decrease pSer^473^Akt in HUVECs in a time-dependent manner. No changes were observed in the total amount of the Akt protein. **(C)** incubation of HUVECs for 16 h with PI3K inhibitor LY 294002 induces phosphorylation of Ser^33^/Ser^37^/Thr^41^ β-catenin in HUVECs. β-actin was used as a loading control.

Several studies have shown that AM acts through PI3K, Akt, MAPK, FAK and other components of the cell-cell adhesion machinery [38–40], suggesting that the PI3K/Akt signalling pathway might be inhibited by AM blockade to produce phospho-Ser^33^/Ser^37^/Thr^41^ β-catenin. Accordingly, we further examined whether αAM or αAMR inhibits 2% FBS-mediated Akt phosphorylation. The incubation of 2% FBS-stimulated endothelial cells with αAM or αAMR did not affect the phosphorylation of Akt at Ser^473^ during the first hours of treatment (Figures 7A, 7B). However, αAM or αAMR decreased the pAkt-Ser^473^ levels after a 10-hour incubation and reached low levels upon a 24-hour incubation (Figures 7A, 7B). This reduction was clearly related to a decrease in phosphorylation, since the total amount of Akt was not altered by αAM nor αAMR treatment (Figures 7A, 7B). We showed that treatment of HUVECs for 16 hours, with PI3K inhibitor LY294002 induced a phosphorylation of Ser^33^/Ser^37^/Thr^41^ β-catenin (Figure 7C). Thus, signalling via a PI3K-Akt-dependent pathway could be involved in AM-CLR-RAMP2/RAMP3 mediated angiogenesis and vascular stability.

### αAM and αAMR promote phosphorylation of β-catenin in nascent tumor vasculature *in vivo*

**Figure 8:**
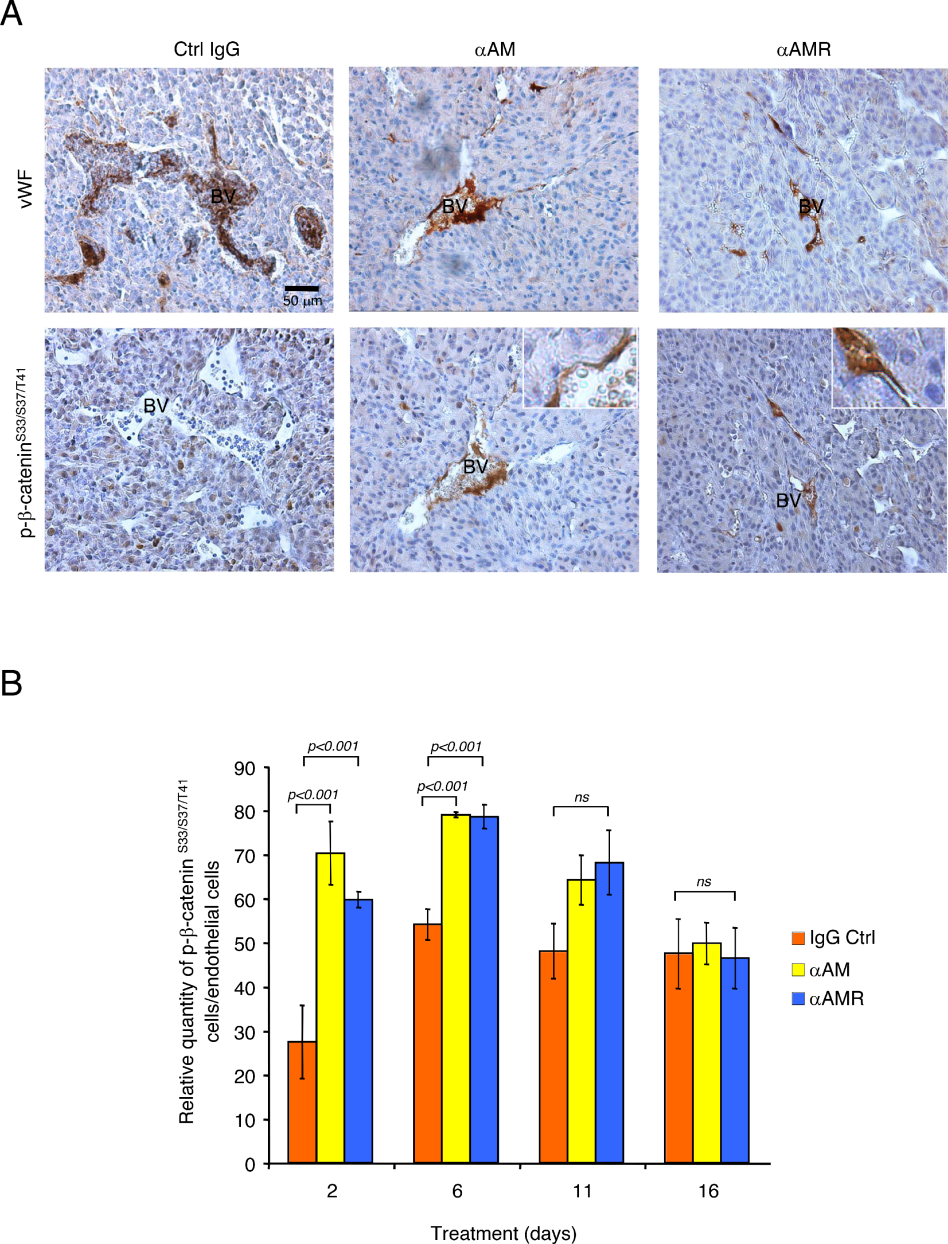
αAM and αAMR induce phosphorylation of β-catenin of vascular endothelial cells in U87 xenografts U87 cells (2.5 × 10^6^) were implanted s.c. into athymic (*nu*/*nu*) mice. After 2, 6, 11, and 16 days of treatment with αAM, αAMR or IgG control, animals were sacrificed and tumor xenografts were harvested. **(A)** microphotographs of serial sections from αAM, αAMR and IgG-control tumors treated for 6 days, were immunostained with vWF and pSer^33^/Ser^37^/Thr^41^ β-catenin antibodies. Sections showed a positive staining for vascular endothelial cells using vWF and pSer^33^/Ser^37^/Thr^41^ β-catenin antibodies. BV; Blood Vessels. **(B)** quantitative assessment of cell density of endothelial cells that stained positive for pSer^33^/Ser^37^/Thr^41^ β-catenin was conducted through microscope. MBF_Image J 1.43 U software was used for analysis. Values are mean ± SE (*n* = 6). Where indicated, statistical analysis was performed with 1-way ANOVA followed by PLSD test, and the level of significance was set at *P* < 0.05.

To determine whether αAM and αAMR treatment of U87 tumor xenografts can induce phosphorylation of the β-catenin in endothelial cells of nascent vessels, groups of animals bearing U87 xenografts were treated i.p. with αAM or αAMR and sacrificed at different times. Histological examination of U87 tumors removed from animals after αAM and αAMR treatment for 2, 6, 11, and 16 days showed a markedly decreased vessel density when compared with the control IgG-treated group. Immunostaining analysis of serial sections of tumors after 6 days of treatment with vWF antibody demonstrates a clear disruption of blood vessels in tumors after 6 days treatment with αAM and αAMR (Figure 8A). In contrast, stable vascularisation can be observed in IgG-control treated tumors (Figure 8A).

**Figure 9:**
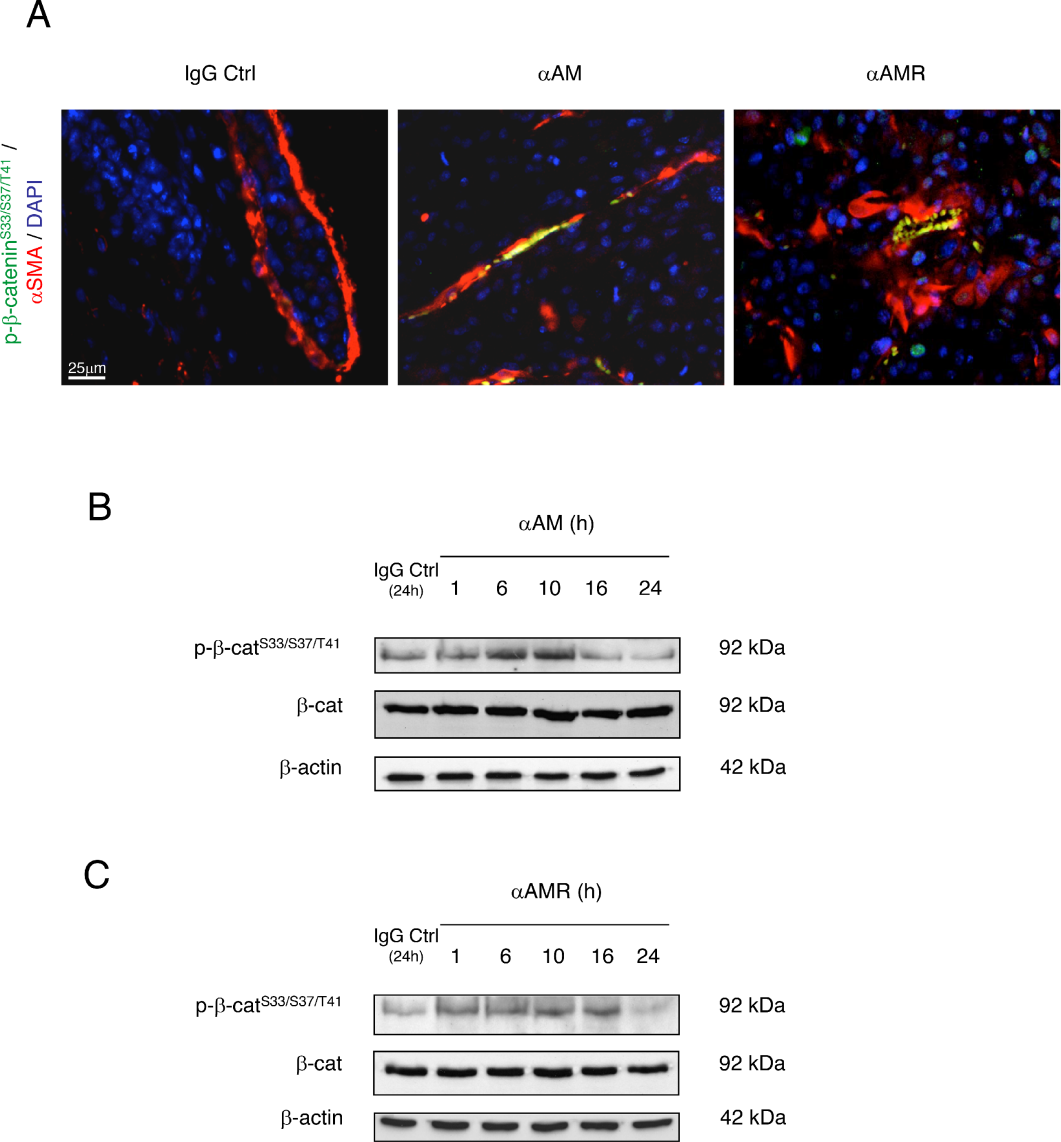
αAM and αAMR induce phosphorylation of Ser^33^/Ser^37^/Thr^41^ β-catenin in pericytes *in vivo* and in HUVSMCs *in vitro* **(A)** tumor sections were evaluated by immunfluorescence for α-SMA (red) and pSer^33^/Ser^37^/Thr^41^-β-catenin (green) using anti-α-SMA and anti**-**pSer^33^/Ser^37^/Thr^41^-β-catenin antibodies in IgG-control, αAM, and αAMR-treated animals for 6 days. DAPI-stained nuclei are in blue. **(B & C)** western blot analysis showed that incubation of HUVSMCs *in vitro* for the indicated times with αAM and αAMR induces pSer^33^/Ser^37^/Thr^41^ β-catenin. β-actin was used as a loading control.

Sections of αAM and αAMR-treated tumors showed vascular endothelial cells that stained with phospho-β-catenin antibody (Figure 8A). No staining for phospho-β-catenin can be observed in vascular endothelial cells in IgG-control section (Figure 8A). Quantification of phospho-β-catenin stained endothelial cells demonstrates a significant increase in αAM and αAMR-treated tumors after 2 and 6 days treatment when compared with IgG-control treated tumors (Figure 8B). No staining differences can be observed at days 11 and 16 that might be the results of phospho-β-catenin degradation after ubiquitination and proteasomal degradation (Figure 8B). Positive phospho-β-catenin staining can be observed for pericytes (α-SMA positive cells) (Figure 9A). Treatment of HUVSMC *in vitro* with αAM and αAMR induces phosphorylation of β-catenin at Ser^33^/Ser^37^/Thr^41^ (Figures 9B, 9C).

**Figure 10:**
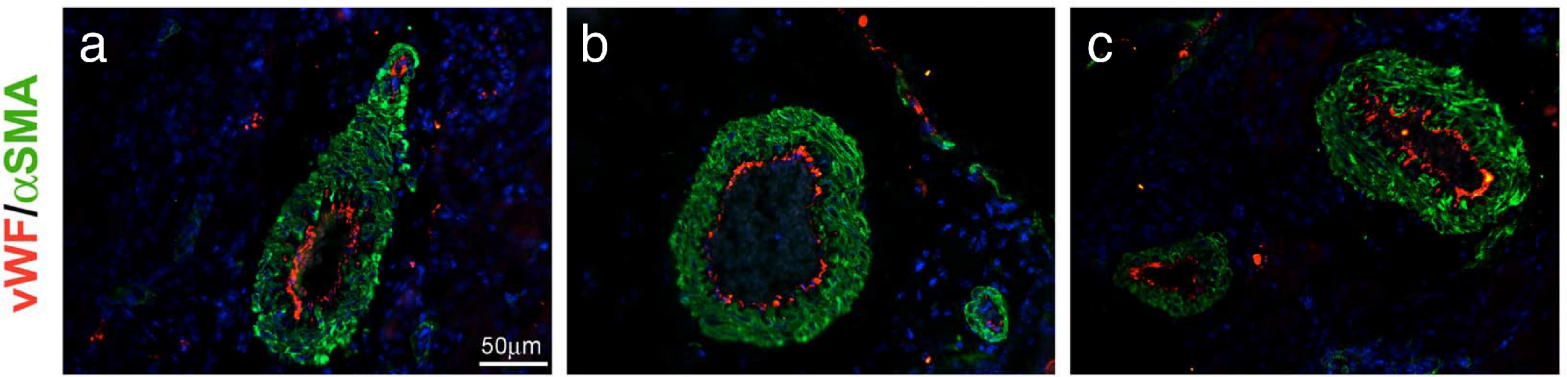
Normal vascularization is not disrupted by αAM and αAMR treatment Representative images of vascular vessels of kidney tissues from control **(a)**, αAM **(b)**, and αAMR **(c)**-treated animals are shown. To visualize functional blood vessels in kidneys, sections (6 μm) were evaluated by immunofluorescence for vWF (red), a marker to detect endothelial cells, and α-SMA (green), a marker for mature pericytes. DAPI-stained nuclei are blue.

It was of interest to observe that AM system blockade did not disrupt the normal vasculature of different tissues in animals bearing the tumor. Sections of kidney tissue from αAM and αAMR-treated mice were stained for endothelial cells and mature pericytes and displayed a normal vasculature suggesting that the treatment was not toxic for the non–tumoral vessels (Figure 10a, 10b, 10c).

## DISCUSSION

Endothelial cells undergoing remodelling or participating in neovessel assembly are in a dynamic state during tumor neoangiogenesis and are thus not firmly attached to the extracellular matrix or to peri-endothelial cells, such as pericytes or vascular smooth muscle cells (SMCs). AM blockade using αAM or αAMR exerts an anti-vascular and antiangiogenic effect presumably by taking advantage of the relative instability of tumor vasculature and its supporting structures, thereby inducing a collapse and regression of tumor neovessels [13–17]. However, the molecular mechanisms induced by αAM and αAMR to exert their selective anti-vascular effects on tumor neovessels are unknown. Here, we show that αAM and αAMR selectively target unstable tumor neovessels through rapid disengagement of the VE-cadherin/β-catenin complex, destabilization of cytoskeleton organization of endothelial cells, and subsequent apoptosis-mediated cell death. In agreement to our data, Sackett *et al*, found that, besides their extracellular behaviour, AM and proadrenomedullin N-terminal 20 peptide (PAMP) can be found in close association with the microtubules in specific cell types [41, 42]. In addition, genetic modifications resulting in the knockdown or knockout of the gene result in morphological changes of the cytoskeleton and in modifications of microtubule-mediated functions such as partial cell cycle arrest, and lower migration capabilities [41].

The different types of blood vessels develop through the assembly of two principal cell-types endothelial cells and pericytes or vascular SMC. Endothelial cells first form tubes, which subsequently recruit a pericytes/SMC coating. Pericytes associated with tumor vasculature contribute to the stability of the capillary wall by participating in the assembly of the basal lamina beneath the endothelial layer, and they also appear to regulate endothelial cell function by, for example, promoting endothelial survival [43]. Our previous study demonstrated that numbers decreased in αAMR treated-tumor xenografts making the vascular endothelial cells more vulnerable to αAMR therapy [17]. These findings suggest that tumor neovessels, lacking proper periendothelial support, are more sensitive to the disrupting actions of αAM and αAMR through VE-cadherin and β-catenin disengagement. This was confirmed by the absence of any toxicity of αAM and αAMR on the stable normal vasculature as shown in this study. The non-disruption of the normal vasculature after AM system blockade suggests that the AM system might be discrete and active at its lowest level in normal stabilized vessels.

AM system blockade disrupts VE-cadherin/β-catenin complex causing inhibition of various endothelial cell vital functions. Incubation of endothelial cells with αAM, αAMR, or AM_22–52_ initiates a series of profound morphological alterations that affect the integrity of cell shape causing anchorage loss, dysregulation of cell-cell interaction that impairs survival signals and leads to cell death by apoptosis with sustained and continuous treatment. AM blockade disrupts endothelial cell-cell contacts which induce an increase of endothelial cell permeability, preventing establishment of cellular connections that are critical for their survival and morphological integrity. The rise/fall effect in Trypan blue-BSA permeability over the treatment time course of 1–30 h, might be explained by the transient inhibition of cell-cell contacts caused by only one treatment of the antibodies and for which the effect starts to disappear by the time due to half-life effects of the antibodies. Greater levels of TB-BSA permeability might be induced and sustained with chronic exposure to the antibodies. Collectively, these data suggest that AM blockade targets tumor neovessels by selective and efficient disruption of vascular endothelial specific VE-cadherin/β-catenin complex.

In support of this conclusion, we demonstrate herein that αAM and αAMR induce first the phosphorylation of Tyr^731^ site which is unique to VE-cadherin, suggesting that regulation of this residue may involve an endothelium-specific mechanism, as would be required for vascular permeability response. Second, the phosphorylation of Ser^33^/Ser^37^/Thr^41^ of β-catenin that targets β-catenin for ubiquitination and proteasomal degradation [33, 34] is therefore interfering with the maintenance of a functional endothelial cell barrier.

Src family kinases activation has been shown to promote uncoupling of VE-cadherin and E-cadherin. We demonstrate that AM blockade induces activation of Src kinase by phosphorylation on Tyr^416^, supporting a putative role for Src in the phosphorylation on Tyr^731^ of VE-cadherin [44]. Inhibition of Src with SU6656 impaired VE-cadherin phosphorylation in αAM- and αAMR-treated HUVECs, indicating that VE-cadherin phosphorylation is dependent on Src activation in this model. Furthermore, inhibition of AM system demonstrates an increased VE-cadherin/Src association likely to better promote VE-cadherin phosphorylation. VE-cadherin may serve as an anchor to maintain Src at the endothelial cell junction, where it could exert its activity on junctional components. Src kinases are considered to play a general role in regulating cadherin function in a wide variety of cell types [45]. In fact, Src can phosphorylate E-cadherin, causing epithelial cells to dissociate from one another [45]. In addition to kinases, phosphatases such as VE-PTP, PTP1B, PTPμ, and SHP-2 have been shown to influence cadherin function [44–48]. For example, VE-PTP is known to associate with VE-cadherin and control its level of phosphorylation [49, 50]. Further studies are needed to determine whether there are any relations between AM system and PTPs.

VE-cadherin and β-catenin chemically cross-link with PI3K and support the survival of endothelial cells and the development of new capillaries in a converging Akt signalling pathway [21]. The fact that αAM and αAMR decreased the pAkt-Ser^473^ supports the concept that VE-cadherin/β-catenin inhibition and PI3K/Akt blockade by αAM or αAMR blocks tumor neovessel assembly and stability. In a similar way, the microtubule-disrupting agent Combretastatin A4 phosphate (CA4P) has been shown to induce rapid regression of tumor neovessels and growth through interference with VE-cadherin signalling [51].

Previous work demonstrated that functional inhibition of adherens junctions using the VE-cadherin mAb BV13 could potently inhibits angiogenesis and tumor growth [52]. However, given the critical role of VE-cadherin in maintaining the integrity of endothelial cells, treatment of mice with BV13 at elevated doses resulted in disruption of adherens junctions in normal tissue vasculature, most notably that of the lungs, leading to an increase in vascular permeability and pulmonary edema [52, 53]. A more selective targeting/blockade of VE-cadherin function on tumor vasculature is an essential requirement for consideration of VE-cadherin as a therapeutic target. Disrupting VE-cadherin/β-catenin complex in tumor neovessels by targeting AM system with αAM and αAMR, with the absence of any toxicity on normal vasculature makes of AM and AMR useful therapeutic targets.

Phosphorylation of Ser^33^/Ser^37^/Thr^41^ β-catenin and the endothelial cell apoptosis observed after treatment on HUVECs *in vitro* and on murine microvessels of tumor xenografts *in vivo*, support strongly that αAM and αAMR might act in the same manner on murine endothelial cells and HUVECs. However, further studies using endothelial cells from a mouse source are needed to compare the effects of αAM and αAMR on mouse and human endothelial cells *in vitro*.

Also, unlike the tumor-associated endothelial cells which are located in microvessels, HUVECs are endothelial cells derived from a macrovessel. Despite their advantage of being a good and convenient model for angiogenesis assays and cell surface interactions studies, HUVECs have been shown to respond differently to signals such as TNF or adrenaline as compared to the human microvascular cells HMECs [54, 55]. In our study, HUVECs and HMECs seem to respond in the same manner after incubation with αAM and αAMR which induced a clear homotypic cell contacts separation, suggesting that the AM system might be important for both endothelial cell types to keep functional interactions and contacts via junction proteins. Experiments to decipher the molecular mechanisms involved in HMECs model are underway.

Taken together, our data suggest that the mechanisms by which αAM and αAMR exert their anti-angiogenic and anti-vascular effects is through rapid functional inhibition of the VE-cadherin/β-catenin complex as needed for several processes such as: endothelial cell-cell adhesion, survival during neovessel assembly, remodelling and disruption of pericyte-endothelial cell association. αAM and αAMR may also inhibit angiogenesis by Akt inactivation, which could interfere with endothelial cell proliferation, gene expression, and tube formation. These findings demonstrate a novel mechanistic insight into the potential effects of αAM and αAMR on the reduction of neoangiogenesis and tumor growth.

## METHODS

### Cell culture

HUVECs, HMECs and HUVSMCs (LONZA) were cultured in EGM-2 medium (LONZA) containing 2% FBS and M199 medium (Invitrogen Life Technologies Inc.) containing 20% FBS, respectively, in humidified incubator at 37°C with air/5% CO_2_. HUVECs and HUVSMCs monolayers from passages 2–4 were used in these studies.

### Capillary tube formation on a matrigel matrix

The morphogenesis assay on Matrigel was performed as previously described [29]. For tube formation, HUVECs (3 × 10^4^) stained with green fluorescent cell linker PKH27 (Sigma-Aldrich) were seeded on Matrigel matrix (Becton Dickenson, Paris, France), and the effect of AM (10^−7^ M), αAM (70 μg/ml), or IgG control (70 μg/ml) (*n* = 3 in duplicates) was analyzed after a 6 h.

### Endothelial cell permeability assay

HUVECs were seeded at a density of 1 × 10^5^ cells in 200 μl/ml onto 6.5 mm diameter on fibronectin-coated transwell filters with a pore size of 0.4 mm (Corning Costar Transwells). Permeability of monolayers was measured in terms of Trypan Blue/BSA (TB-BSA) transfer according to McQuaid *et al*. [56]. Endothelial cells medium was changed every 24 h until confluence, then was replaced by medium supplemented with or without AM (10^−7^ M) (Bachem), rabbit IgG anti-AM antibody (αAM; 70 μg/ml), rabbit IgG anti-AMR antibodies (αAMR; 70 μg/ml), or rabbit IgG control (70 μg/ml) for 1, 6 and 24 h. αAM and αAMR were developed in our laboratory [13, 17]. For the assay, membranes were incubated with test ligands in 150 μl of Hank's balanced salt solution with 30 mM Hepes (HBSS) with TB-BSA (66 kDa; Sigma) at a final concentration of 40 μg/ml in the upper chamber. Lower chamber contained 600 μl of HBSS; incubation was performed for 90 min in presence of 5% CO2 with gentle shaking at 37°C. Optical density of the lower chamber solution was measured at 570 nm. The relative permeability was calculated by dividing the OD of treated samples by vehicle control.

### Fluorescence microscopy analysis

The fluorescence microscopy analyses of VE-cadherin, β-catenin, and phospho-β-catenin Ser^33^/Ser^37^/Thr^41  ^were performed on the HUVECs in different conditions and times as indicated in Results. Briefly, after cells were fixed in 4% paraformaldehyde and permeabilized with 0.1% Triton X-100, cells were incubated with polyclonal antibodies against VE-cadherin (1:100; BD Transduction Laboratories), β-catenin (1:100; BD Transduction Laboratories), and phospho-β-catenin Ser^33^/Ser^37^/Thr^41^ (1:50; Cell Signaling Technology) overnight at 4°C, then washed and incubated with secondary Alexa Fluor–conjugated antibodies (1:300; Vector Laboratories) for 45 min at room temperature. After washing, the samples were mounted in VECTASHIELD (Vector Laboratories) and analyzed by fluorescence microscopy.

### Western blot analysis

HUVECs (2 × 10^6^) or HUVSMCs (2 × 10^6^) were incubated under different conditions and for various times as indicated in Results after an overnight starvation with X-VIVO medium. Cell extracts were prepared and processed for Western blot analysis as described [15]. Binding of the primary antibody against polyclonal VE-cadherin (1:1,000; Cell Signaling Technology), polyclonal VE-cadherin phospho-Tyr^731^ (1:500; Invitrogen Life Technologies), polyclonal β-catenin phospho-Ser^33^/Ser^37^/Thr^41^ (1:1,000; Cell Signaling Technology), polyclonal β-catenin (1:500; Santa Cruz Biotechnology Inc.), polyclonal Akt or polyclonal Akt phospho-Ser^473^ (1:1,000; Cell Signaling Technology), polyclonal Src or polyclonal Src phospho-Tyr^416^ (1:1,000; Cell Signaling Technology), polyclonal β-actin (1:1,000; Cell Signaling Technology) was detected with the enhanced chemiluminescence reagent (ECL kit, Invitrogen Life Technologies Inc.) using HRP-conjugated secondary antibody (1:3,000; Dako Cytomation).

### Immunoprecipitation and western blotting

HUVECs (3 × 10^6^) were incubated in different conditions and times as indicated in Results after an overnight starvation with X-VIVO medium. Cells were lysed in 0.5 ml ice-cold RIPA buffer containing protease inhibitors and 1 mM Na_3_VO_4_ as described [37]. The protein concentration of the cell lysate was then determined, and antibody against VE-cadherin (4 μg; Santa Cruz Biotechnology Inc.) was added to the same amount of protein lysate and incubated for 2 h at 4°C. The protein G agarose beads (100 μl) (Pierce Biotechnology Inc.) was used to capture the immune-complex for 1 h, and then beads were sediment by brief centrifugation and washed by suspending and pelleting 3 times with 0.5 ml ice-cold modified RIPA buffer. Then, the agarose beads were suspended in 60 μl sample buffer, boiled for 5 minutes, and collected by centrifugation, and 20 μl of the supernatant fraction was fractionated on 8% SDS-polyacrylamide gel and processed for immunoblotting with anti-VE cadherin, anti-Src, and anti-Src phosphoTyr^416^ antibodies as above. Representative data from at least three separate experiments are shown.

### *In vivo* tumor growth assessment

Athymic NMRI (*nu*/*nu*) nude mice were implanted with U87 glioblastoma cells. Cell suspension of 2.5 × 10^6^ cells was injected s.c. as described [13]. Two weeks later, most tumors had grown to 300–500 mm^3^, and mice were randomized into groups. Three independent experiments were performed, each totalizing 10 animals in 3 groups. The treatment was i.p. injection, received every 3 days, of preimmune serum (12 mg/kg of purified IgG), αAMR (12 mg/kg of purified IgG/mouse) [17], and αAM (12 mg/kg of purified IgG/mouse) [13, 17]. The body weight, tumor size, and general clinical status were recorded every 3 days as described [17].

### Immunohistochemical analysis

All tumors were excised, fixed in 4% (vol/vol) formalin, and processed for immunohistochemical analysis. Paraffin blocks were cut to 6-μm sections and stained with H&E for morphology evaluation. Immunohistochemistry was carried out using the Vectastain Elite ABC Kit (Vector Laboratories). Thin (6-μm) sections were incubated with anti-von Will brand factor (1:400; Dako), anti-α-SMA (1:80; Dako), phospho-β-catenin Ser^33^/Ser^37^/Thr^41^ (1:50; Cell Signaling Technology) antibodies, and subsequently with fluorochrome (Alexa 488 or Alexa 562)-conjugated secondary antibodies (Invitrogen Life Technologies). A programmed cell death was evaluated using mAb F7–26 to detect single-strand DNA (1:100; AbCys, Paris, France). For non-immunofluorescence staining, detection was carried out using a DAB chromogen. Negative control slides were obtained by omitting the primary antibody.

### Statistical analyses

Data are expressed as mean ± SEM. Statistical analyses were performed using the 1-way ANOVA followed by Fisher's protected least significant difference (PLSD) test (Statview 512, Brain Power Inc., Calabasas, CA, US). The difference was considered significant at values of *P* < 0.05.

## SUPPLEMENTARY FIGURE


