# Supplementary material for: Adrenomedullin blockade induces regression of tumor neovessels through interference with vascular endothelial-cadherin signalling
Source: Oncotarget. 2015 Feb 5;6(10):7536–53. doi: 10.18632/oncotarget.3167 (PMC4480698; doi:10.18632/oncotarget.3167)
Supplement: Supplementary file 1 [file oncotarget-06-7536-s001.pdf]

SUPPLEMENTARY FIGURE

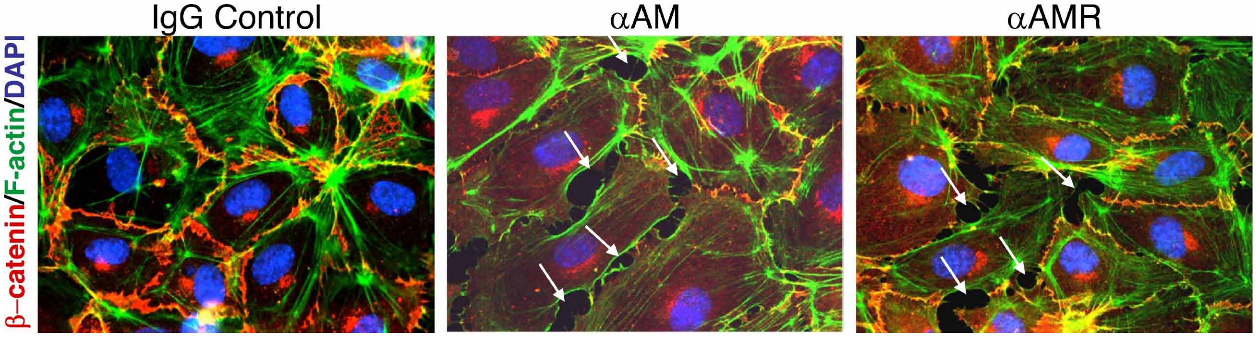

Supplementary Figure S1: αAM and αAMR disrupt cell-cell contacts of HMECs *in vitro*. αAM and αAMR treatment for 10 h caused a disengagement between cells, a reorganisation of actin fibers stained with phalloidin (green) that become localized around the cell body and β-catenin staining observed in the cytoplasm when compared to IgG-control treated cells. DAPI-stained nuclei are in blue.
